# Predictors of suboptimal CD4 response among women achieving virologic suppression in a randomized antiretroviral treatment trial, Africa

**DOI:** 10.1186/1471-2334-14-331

**Published:** 2014-06-17

**Authors:** Aida Asmelash, Yu Zheng, Kara Wools Kaloustian, Douglas Shaffer, Fred Sawe, Anthony Ogwu, Robert Salata, Judith Currier, Michael D Hughes, Shahin Lockman

**Affiliations:** 1 Botswana Harvard School of Public Health AIDS Initiative Partnership, Gaborone, Botswana; 2 Harvard School of Public Health, Boston, MA, USA; 3 Brigham and Women’s Hospital, Boston, MA, USA; 4 University of California, Los Angeles, LA, USA; 5 Case Western Reserve University, Cleveland, OH, USA; 6 Indiana University, Indianapolis, USA; 7 Walter Reed Project, Kericho, Kenya

**Keywords:** HIV, Antiretroviral therapy, HAART, Immune response, CD4

## Abstract

**Background:**

A subset of HIV-1 infected patients starting highly active antiretroviral treatment (HAART) experience suboptimal CD4 response (SCR) despite virologic suppression. We studied the rate of and risk factors for SCR among women starting HAART in the ACTG A5208 study conducted in 7 African countries. 741 HAART-naive women with screening CD4 count <200 cells/μL were randomized to start HAART with Tenofovir/Emtricitabine plus either Nevirapine or Lopinavir/Ritonavir.

**Methods:**

This analysis includes the 625 women who remained on-study through 48 weeks without experiencing protocol-defined virologic failure. We defined SCR as < 100 CD4 cells/μL increase from baseline and absolute CD4 cell count < 350 cells/μL, both at 48 weeks after HAART initiation.

**Results:**

The baseline characteristics for the 625 women prior to HAART initiation were: median age 33 years, screening CD4 count 134 cells/μL, and HIV-1 RNA 5.1 log_10_ copies/mL; 184 (29%) were WHO Stage 3 or 4.

Seventy one (11%) of these 625 women experienced SCR. Baseline factors independently associated with increased odds of SCR included older age, lower HIV-1 RNA, positive Hepatitis B surface antigen, and site location. At 96 weeks, only 6% of the SCR group had CD4 ≥ 350 cells/μL compared with 67% in the non SCR group.

**Conclusion:**

After starting HAART, 11% of women with virologic suppression through 48 weeks experienced SCR. These patients were also less likely to achieve CD4 ≥ 350 cells/μL by 96 weeks. The underlying causes and long term clinical implications of SCR deserve further investigation**.**

**Trial registration:**

Clinicaltrials.gov Identifier: NCT00089505

## Background

Highly active antiretroviral therapy (HAART) is effective in decreasing the morbidity and mortality associated with HIV-1 infection by reducing HIV-1 replication, which enables increases in CD4 cell count and helps to restore immune function [1-6]. However, in some patients with HIV-1 infection, a substantial proportion (10-30%) of individuals show incomplete or poor CD4 cell recovery on HAART despite suppression of HIV-1 to undetectable levels [4].

Several studies have evaluated factors associated with suboptimal CD4 restoration in HIV −1 infected patients taking HAART [1-10]. Results of studies which looked at predictors of discordant responses (with virologic suppression but incomplete CD4 recovery) have been varied. Furthermore, data evaluating predictors of SCR in resource limited settings and among women are limited.

We undertook an analysis of risk factors predicting SCR among women initiating HAART in 7 countries in Africa who were enrolled in a randomized treatment trial. We defined SCR (prior to analyses) as an increase of < 100 cells/μL in CD4 count from baseline, and an absolute CD4 cell count of < 350 cells/μL, both at 48 weeks after HAART initiation, among women with virologic suppression throughout the 48 weeks. This definition of SCR was selected due to a combination of a) its clinical relevance, based on knowledge of significantly higher rates of HIV-associated morbidity and mortality associated with CD4 < =350 cells/μL compared with >350 cells/μL, and b) a conservative expectation (based on prior experience/literature) that the majority of patients starting ART with CD4 < 200 cells/μL should experience CD4 increase of >100 cells/μL and achieve CD4 > 350 cells/μL by 48 weeks after treatment start.

## Methods

This secondary analysis used data from the ACTG A5208/OCTANE study. The design and primary results of the OCTANE study have been previously published [11]. In brief, study participants were 741 women with screening CD4 count <200 cells/μL who were antiretroviral-naïve except for prior single dose Nevirapine exposure (SD NVP) in a subset of 241 participants. Prior Zidovudine use for up to 10 weeks for the prevention of mother-to-child transmission of HIV-1 was not exclusionary. Participants were enrolled in 7 countries in Africa (South Africa, Botswana, Zimbabwe, Kenya, Malawi, Zambia, and Uganda). Individuals with active tuberculosis disease or severe illness requiring systemic therapy or hospitalization were ineligible, as were pregnant women. All participants were randomized 1:1 to start open-label HAART with either Lopinavir/Ritonavir plus Tenofovir/Emtricitabine (LPV/r + TDF/FTC) or with Nevirapine plus Tenofovir/Emtricitabine (NVP + TDF/FTC). Participants were followed until the last woman to enroll had completed 48 weeks of follow-up.

CD4 cell count and HIV-1 RNA level, clinical assessments, hematology and chemistry tests were evaluated at screening, baseline (before HAART initiation) and at 4, 12, 16, and 24 weeks on study and then every 12 weeks. Adherence was evaluated through self reported missed dose in the past 3 days at week 4, 12, 24 and 48. The study defined adherence by week 48 as not reporting any missed dose at or before week 48.

Only participants who remained in follow-up on the study through to at least 48 weeks and who did not experience protocol-defined virologic failure at or before week 48 were included in this analysis of SCR. Virologic failure was defined as a confirmed HIV-1 RNA decrease of < 1 log_10_ copies/mL from baseline at week 12, or a confirmed HIV-1 RNA ≥400 copies/mL at or after week 24 irrespective of change in HAART regimen.

Although the screening CD4 cell count had to be 200 cells/μL or less within 90 days of enrollment there was no restriction on the baseline CD4 cell count (obtained from a sample drawn at the time of treatment initiation). 47 women had screening CD4 count <200 cells/μL but enrollment CD4 count >200 cells/μL.

### Statistical methods

SCR was defined as an increase of < 100 cells/μL in CD4 count from baseline, and an absolute CD4 cell count of < 350 cells/μL, both at 48 weeks after HAART initiation, among women with virologic suppression throughout the 48 weeks. Non-SCR was defined as..an increase of ≧ 100 cells/μL in CD4 count or CD4 cell count of ≥350 cells/μL.

The association between the following baseline variables and SCR within the first 48 weeks of treatment were evaluated in both univariate tests (Wilcoxon rank sum test for continuous variables, Fisher’s exact test for categorical variables, and Cochran-Armitage trend test for WHO stage) and multivariate logistic regression analysis: age, enrollment site, CD4 cell count, HIV-1 RNA, body mass index, hepatitis B co-infection status, WHO stage, prior exposure to SD NVP, and assigned HAART regimen. The association between SCR in the first 48 weeks of treatment and the following post-baseline factors was also evaluated: adherence to HAART by self report, and the occurrence of potential HIV/AIDS-related diagnoses within the first 48 weeks and after 48 weeks on-study. The selection of covariates in the final multivariate models was done by stepwise selection requiring P < 0.05 for inclusion/retention of a variable, except that screening CD4 count was forced into the model to take account of the possibility that women with lower screening counts might be less likely to achieve the threshold of 350 cells/μL by week 48. A P-value of <0.05 was considered statistically significant. The inclusion/retention criteria for stepwise selection refer to the threshold for forward and backward selection. This procedure selects significant variables adjusted for other variables.

The protocol and informed consent was reviewed and approved by the following ethics committees at each site: Medicines Control Authority of Zimbabwe, South Africa Johannesburg - WITS - Human Research Ethics Committee: Medical (HREC), Kenya Kericho - Kenya Medical Research Institute (KEMRI) ERC & Walter Reed Army Institute of Research (WRAIR),Malawi Lilongwe – NHSRC, South Africa Soweto - Wits HREC, Kenya Eldoret - Human Research Protections Office (HRPO) & National Council for Science and Technology (NCST), Zambia Lusaka - University of Zambia Biomedical Research Ethics Committee (UNZA BREC), Uganda Kampala – Joint Clinical Research Center IRB; Uganda National Council for Science and Technology, South Africa Durban - Biomedical research Ethics committee (BREC), Botswana Human Research Unit, and Office of Human Research Administration at Harvard School of Public Health.

All study participants signed an informed consent before study participation. The manuscript was reviewed and signed off by all authors.

## Results

Six hundred and twenty five women without protocol-defined virologic failure through 48 weeks were included in the analysis. Baseline characteristics were: median age 33 years, median screening CD4 count 134 cells/μL, and median HIV-1 RNA 5.1 log_10_ copies/mL; 184 (29%) women were at WHO Stage 3 or 4 (Table 1). Seventy-one (11%) of these 625 women experienced SCR, while 554 (89%) were non SCR. Of the expected cumulative CD4 results only 2.2% were missed or not obtained during follow-up in the study.

**Table 1:** Baseline characteristics of the 625 study participants (who did not experience protocol defined virologic failure through to week 48), and univariate associations with suboptimal CD4 response

**Characteristics**		**All women without virologic failure through week 48 (N = 625)**	**SCR (N = 71)**	**Non-SCR (N = 554)**	**Univariate Pvalue**
Age (years)	Median (IQR^1^)	33 (28, 38)	37 (31, 41)	32 (28, 37)	<.001
Screening CD4 (cells/μL)	Median (IQR)	134 (88, 168)	127 (80, 163)	135 (90, 168)	0.38
Baseline CD4 (cells/μL)	Median (IQR)	132 (88, 177)	141 (104, 193)	130 (85, 174)	0.08
	<50	63 (10%)	3 (4%)	60 (11%)	
	> = 50	562 (90%)	68 (96%)	494 (89%)	
HIV-1 RNA (copies/ml)	Median (IQR)	5.13 (4.72, 5.53)	5.00 (4.29, 5.41)	5.15 (4.77, 5.53)	0.005
CD4/CD8 ratio	Median (IQR)	0.17 (0.10, 0.25)	0.19 (0.11, 0.26)	0.17 (0.10, 0.25)	0.48
BMI (kg/m^2^)	Underweight:<18.5	58 (9%)	6 (8%)	52 (9%)	0.18
	Normal: 18.5- < 25	372 (60%)	48 (68%)	324 (58%)	
	Overweight: 25- < 30	123 (20%)	14 (20%)	109 (20%)	
	Obese: > = 30	72 (11%)	3 (4%)	69 (12%)	
Hepatitis B surface antigen	Positive	35 (6%)	7 (10%)	28 (5%)	0.10
	Negative	587 (94%)	64 (90%)	523 (95%)	
	Missing	3	0	3	
WHO stage	I	254 (41%)	36 (51%)	218 (39%)	0.02
	II	187 (30%)	22 (31%)	165 (30%)	
	III/IV	184 (29%)	13 (18%)	171 (31%)	
SD NVP exposure prior to study entry	Yes	204 (33%)	17 (24%)	187 (34%)	0.11
	No	421 (67%)	54 (76%)	367 (66%)	
Initial randomized regimen	NVP	298 (48%)	38 (54%)	260 (47%)	0.32
	LPV/r	327 (52%)	33 (46%)	294 (53%)	
Site	Botswana	81 (13%)	8 (11%)	73 (13%)	0.001
	Durban (SA^2^)	44 (7%)	5 (7%)	39 (7%)	
	Eldoret(Kenya)	54 (9%)	16 (23%)	38 (7%)	
	Kericho(Kenya)	70 (11%)	6 (8%)	64 (12%)	
	Jo’burg^3^(SA^2^)	56 (9%)	2 (3%)	54 (10%)	
	Jo’burg^3^ (Wits^4^)(SA^2^)	74 (12%)	8 (11%)	66 (12%)	
	Malawi	50 (8%)	5 (7%)	45 (8%)	
	Uganda	50 (8%)	6 (8%)	44 (8%)	
	Zambia	55 (9%)	10 (14%)	45 (8%)	
	Zimbabwe	91 (15%)	5 (7%) 86	(16%)	

^1^Inter-quartile range, ^2^South Africa, ^3^Johannesburg, ^4^University of Witwatersrand.

In univariate analysis, patients with SCR were older than patients with non SCR (median age 37 years versus 33 years respectively, P < 0.001), had lower viral load at baseline (5.0 log_10_ copies/mL versus 5.2 log_10_ copies/mL respectively, P = 0.005), and had lower WHO stage (51% versus 39% for stage I and 18% versus 30% for stage III/IV, P = 0.02). There was statistically significant variation in CD4 response among participating sites, with a Kenyan site (Eldoret) having the highest SCR rate (30%) and a Johannesburg site the lowest (4%). Baseline Body mass index, exposure to single dose nevirapine, treatment arm (nevirapine/tenofovir/emtricitabine versus lopinavir/ritonavir/tenofovir/emtricitabine) and adherence were not significantly associated with SCR (P > 0.05). Sixty one percent of women in the SCR group and 67% in the non-SCR group reported missing no doses through week 48 (adherence was not associated with SCR in univariate analysis, P = 0.42).

**Figure 1:**
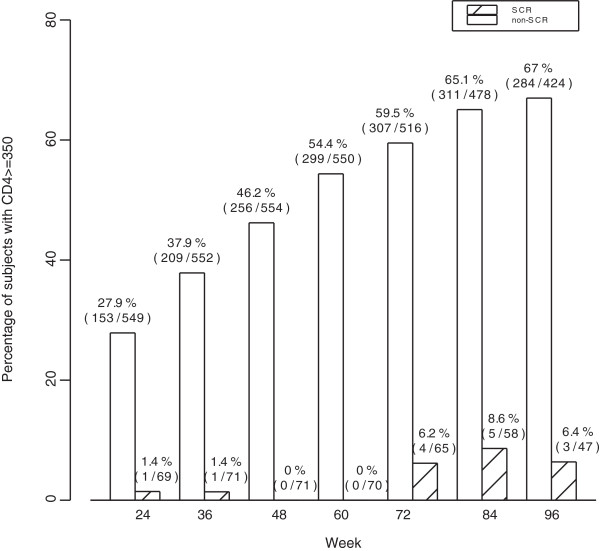
Percentage of women with CD4≥ 350 cells/μL by time among women with SCR vs. non-SCR.

In multivariate analysis, the following variables were significantly associated with increased odds of SCR: older age, lower baseline HIV-1 RNA, positive hepatitis B surface antigen, and enrollment site (Table 2).At 48 weeks, 46% of women in the non-SCR group had CD4 ≥ 350 cells/μL. Among the 47 women in the SCR group followed to 96 weeks, only 6% had CD4 ≥ 350 cells/μL, compared with 67% of the 424 women in the non-SCR group (Figure 1).

**Table 2:** Multivariate analysis of risk factors for suboptimal CD4 response

**Variable**		**Odds ratio (95% CI)**	**P-value for overall effect**
Age	per 10 years increase	2.32 (1.60, 3.34)	<0.001
Baseline HIV-1 RNA	per 1 log10 copies/mL increase	0.46 (0.30, 0.71)	<0.001
Screening CD4	per 100 cells/μL) increase	0.66 (0.38,1.15)	0.14
Hepatitis B surf. antigen	Positive vs. Negative	2.86 (1.05, 7.77)	0.040
Enrollment site	Harare Zimbabwe^1^	1 (reference site)	
	Botswana	2.18 (0.60, 7.89)	0.005
	Durban SA ^2^	2.96 (0.76, 11.57)	
	Eldoret Kenya	9.63 (3.01, 30.82)	
	Kericho Kenya	2.35 (0.63, 8.77)	
	Johannesburg	SA^2^ 1.15 (0.20, 6.55)	
	Johannesburg	WITS^3^ SA^2^ 2.97 (0.83,10.57)	
	Lilongwe Malawi	2.21 (0.55, 8.90)	
	Kampala Uganda	2.77 (0.75, 10.22)	
	Lusaka Zambia	5.05 (1.53, 16.67)	

^1^Reference category ^2^South Africa ^3^University of Witwatersrand.

For the 154 women who did not have week 96 CD4 results, the main reason is study completion prior to week 96 follow-up: 140 (91%) completed the study but were followed less than 96 weeks, 11 were lost to follow-up prior to study completion, and 3 were due to missing visits. Among the 625 women included in the analysis, 25 experienced virologic failure between week 48 and 96 (20 in non-SCR group and 5 in SCR group).

### CD8 and CD4 response

Women with and without SCR on HAART had similar baseline absolute CD8+ cell counts (median 790 cells/μL vs. 702 cells/μL, respectively) and CD4/CD8 ratio (0.19 for SCR vs. 0.17 for non-SCR), although the proportion of women with baseline CD8 ≥ 1200 cells/μL (the upper limit of normal according to clinical laboratory reference values) was somewhat higher in the SCR group (26% vs. 17% respectively, P = 0.07). The SCR group experienced a greater decrease in absolute CD8 count prior to or at week 48 compared to the women with non SCR, CD4 response (median change from baseline −246 vs. +27 cells/μL at week 48, P < 0.001). However, the CD4/CD8 ratio at week 48 was lower in the SCR group than among the non SCR’s (median CD4/CD8: 0.31 versus 0.43 respectively, P < 0.001).

### Clinical outcomes and SCR

There was no significant difference in the incidence of new potentially HIV-1 related clinical diagnoses between women with SCR versus without SCR both during the first 48 weeks following treatment initiation (relative risk [RR] 0.95, P = 0.65) and after 48 weeks (RR 1.19, P = 0.34).

Only 9 (1%) of the 625 women had disease progression to a higher WHO Stage after week 48, with no significant difference among those with versus without SCR (2 [3%] vs. 7 [1%], respectively).

## Discussion and conclusions

In this study among 625 highly immunocompromised African HIV-1 infected women who achieved and maintained virologic suppression through 48 weeks following initiation of first-line HAART, 11% of women experienced SCR. This proportion is consistent with rates reported in other studies, ranging from 10.5% to 19.0% [2-4,7], despite varying definitions used for poor immunologic response.

While it is reassuring that the vast majority of women demonstrated adequate CD4 recovery in this setting, it is concerning that only 6% of patients with SCR achieved CD4 ≥ 350 cells/μL by 96 weeks after treatment initiation. We did not observe a significantly higher rate of adverse clinical outcomes among women with SCR compared with non-SCR but we had limited power to investigate the clinical implications of SCR and a relatively short period of follow-up.

Several patient factors were associated with SCR. One of these factors was older age at HAART initiation; this association has also been observed in other studies [1-5], and is postulated to be related to thymus degeneration [5]. We did not find screening CD4 count to be significantly associated with SCR; one study showed higher baseline CD4 related to SCR in the univariate analysis but was not shown to be an independent risk factor [8], while others found lower baseline CD4 to be a risk factor [9]. The finding that lower baseline HIV-1 RNA was associated with SCR in our participants, while somewhat counter-intuitive, has been observed in other studies [6,10,12]. This phenomenon has been hypothesized to result from possible prior inn ate suppression of HIV replication, and as such a degree of CD4 gain may have occurred already, limiting further increase [5,6]. However, baseline CD4 was similar in both the SCR and non-SCR groups, and lower HIV-1 RNA was associated with SCR even after controlling for screening CD4 in multivariate analysis, suggesting that this mechanism may not fully explain this association.

To our knowledge, the association between HBV infection and SCR has not been previously observed. Some studies have found no significant difference in the virologic and immunologic responses to HAART by hepatitis B co-infection status [1,13]. One study found that HIV and HBV co-infected patients had lower CD4 T cell count at ART initiation, and HBeAg positivity status decreased the likelihood of achieving undetectable HIV load after 24 weeks of ART [14]. There is need for more studies to better understand the role of HBV on the response to ART in HIV/HBV co infected patients.

We did not find an association between baseline body mass index and SCR, in contrast to a recent study which showed that 12-month CD4 lymphocyte recovery was greatest among patients commonly classified as overweight (BMI range of 25–30 kg/m^2^ ) [15].

Rates of SCR among women with virologic suppression differed among sites (4%-30%). The difference between sites could be related to host or virus genetic differences, or other factors like co-infection with other pathogens or the type of nutritional intake.

Patients with SCR are less likely to reach CD4 ≥ 350 cells/μL at 96 weeks, and early SCR may therefore alert clinicians to the possibility that immune failure early on treatment could be a predictor of long term failure.

The strengths of our study included very high rates of follow-up and of completeness of laboratory and other data. The study also involved a large cohort composed entirely of women in Africa, a population severely affected by the HIV-1 epidemic but in whom minimal longitudinal CD4 response data (after HAART initiation) are available. Limitations of our study included use of an HIV-1 RNA assay with a limit of detection of 400 copies/mL (such that participants with HIV-1 RNA replication but levels <400 copies/mL were not identified); and a sample size that was too small to detect potential clinical implications of SCR. Hepatitis C (HCV) status and intravenous drug use (IDU) data was not available and not included in the analysis. Some studies have shown HCV and IDU to be related to level of CD4 response while on therapy [16,17].

Prior studies have established that lack of CD4 reconstitution is associated with greater risk of mortality and morbidity [1-4,18,19]. It is therefore important to understand the scope and underlying causes of the occurrence of SCR to devise approaches to improving immune reconstitution in these patients who are at risk of adverse clinical outcomes even after full virologic suppression on HAART.

## Competing interest

Dr. Hughes reports having previously been a paid member of data monitoring committees for Boeringer Ingelheim, Medicines Development, Pfizer and Tibotec. The other authors have no competing interests.

## Authors’ contributions

SL: Protocol Chair. JC: Protocol Vice Chair. YZ: Statistician. MH: Statistician. KW-K: Investigator. FS: Investigator. DS: Investigator. RS: Investigator. AO: Investigator. AA: Investigator. All authors read and approved the final manuscript.

## Pre-publication history

The pre-publication history for this paper can be accessed here:

http://www.biomedcentral.com/1471-2334/14/331/prepub
